# IL-27-containing exosomes secreted by innate B-1a cells suppress and ameliorate uveitis

**DOI:** 10.3389/fimmu.2023.1071162

**Published:** 2023-06-02

**Authors:** Minkyung Kang, Manoj Kumar Yadav, Evaristus C. Mbanefo, Cheng-Rong Yu, Charles E. Egwuagu

**Affiliations:** Molecular Immunology Section, Laboratory of Immunology, National Eye Institute (NEI), National Institutes of Health (NIH), Bethesda, MD, United States

**Keywords:** exososomes, IL-27, immunotherapy, bregs, uveitis, CNS autoimmune diseases, i27-Exosomes

## Abstract

**Introduction:**

IL-27 is a heterodimeric cytokine composed of Ebi3 and IL-27p28 and can exert proinflammatory or immune suppressive effects depending on the physiological context. Ebi3 does not contain membrane-anchoring motifs, suggesting that it is a secreted protein while IL-27p28 is poorly secreted. How IL-27p28 and Ebi3 dimerize *in-vivo* to form biologically active IL-27 is unknown. Major impediment to clinical use of IL-27 derives from difficulty of determining exact amount of bioavailable heterodimeric IL-27 needed for therapy.

**Methods:**

To understand how IL-27 mediates immune suppression, we characterized an innate IL-27-producing B-1a regulatory B cell population (i27-Breg) and mechanisms i27-Bregs utilize to suppress neuroinflammation in mouse model of uveitis. We also investigated biosynthesis of IL-27 and i27-Breg immunobiology by FACS, immunohistochemical and confocal microscopy.

**Results:**

Contrary to prevailing view that IL-27 is a soluble cytokine, we show that i27-Bregs express membrane-bound IL-27. Immunohistochemical and confocal analyses co-localized expression of IL-27p28 at the plasma membrane in association with CD81 tetraspanin, a BCR-coreceptor protein and revealed that IL-27p28 is a transmembrane protein in B cells. Most surprising, we found that i27-Bregs secrete IL-27-containing exosomes (i27-exosomes) and adoptive transfer of i27-exosomes suppressed uveitis by antagonizing Th1/Th17 cells, up-regulating inhibitory-receptors associated with T-cell exhaustion while inducing Treg expansion.

**Discussion:**

Use of i27-exosomes thus obviates the IL-27 dosing problem, making it possible to determine bioavailable heterodimeric IL-27 needed for therapy. Moreover, as exosomes readily cross the blood-retina-barrier and no adverse effects were observed in mice treated with i27-exosome, results of this study suggest that i27-exosomes might be a promising therapeutic approach for CNS autoimmune diseases.

## Introduction

Interleukin 27 (IL-27) is a member of the IL-12 family of cytokines and is comprised of IL-27p28 and Ebi3 (Epstein-Barr virus (EBV)-induced gene 3) subunits. The IL-27p28 subunit (IL-30) shares structural homology with IL-6 and IL-12p35 while Ebi3 is homologous to members of the class I cytokine receptor family such as IL-6 receptor and IL-12p40 protein (1–4). In contrast to IL-12 that is secreted as a disulfide-linked IL-12p35:IL-12p40 heterodimeric cytokine, IL-27 is not secreted as a disulfide-linked heterodimer (4, 5). IL-27p28 and Ebi3 are thought to be independently secreted proteins that eventually associate *in-vivo* to form the biologically active IL-27 by yet unknown mechanism(s). Although either Ebi3 or IL-27p28 can inhibit lymphocyte proliferation *in-vitro*, co-expression of Ebi3 and IL-27p28 in the same cell is required for production of the bioactive IL-27 cytokine.

IL-27 is structurally and functionally similar to Interleukin 35 (IL-35), another immune-suppressive member of the IL-12 family (6–8). While IL-35 is strictly an immunosuppressive cytokine, IL-27 can be pro- or anti-inflammatory, suggesting that IL-27 and IL-35 regulate immunity by similar as well as distinct mechanisms (9). Consequently, both features of IL-27 have been exploited to promote anti-tumor responses (10, 11) and for treatment of autoimmune diseases (6, 12, 13). In fact, increase of IL-27 in cerebrospinal fluid of patients with multiple sclerosis correlates with suppression of neuroinflammation (14), underscoring therapeutic potential of IL-27 as treatment for human CNS autoimmune diseases.

We recently identified an innate-like B-1a cell population in mouse peritoneal cavity that produces IL-27 and the IL-27-producing B-1a regulatory cells (i27-Breg) suppress experimental autoimmune uveitis (EAU) or experimental autoimmune encephalomyelitis (EAE), two CNS mouse autoimmune disease models of human uveitis and multiple sclerosis, respectively (15). Adoptive transfer of the i27-Bregs suppress uveitis and encephalomyelitis through sustained secretion of IL-27 in retina, brain, spinal cord and lymphoid tissues (15). Interestingly, in a previous study we described an IL-35-producing Breg population (i35-Breg) that suppresses neuroinflammation through its signature cytokine IL-35 (15, 16) and by secreting IL-35-containing exosomes (i35-exosomes) (17). That observation suggests that i27-Bregs may also secrete IL-27-containing exosomes (i27-exosomes). Exosomes are nanosized extracellular vesicles whose constituents generally reflect cytoplasmic content of the parent cell and may contain regulatory proteins, mRNA, miRNA and LncRNA (18, 19). Exosomes secreted by lymphocytes activate diverse immunoregulatory functions and because of their small sizes (50nm-150nm) they are able to cross the blood-brain-barrier or blood-retina-barrier, making them ideal vehicles for therapeutic delivery of immunosuppressive cytokines into CNS tissues (20). It was therefore of interest to investigate whether i27-Bregs may also suppress uveitis in part by secreting i27-exosomes.

In this study, we characterized i27-Breg cells and to our surprise found that they express membrane-bound IL-27 contrary to the common notion that IL-27 is a soluble cytokine. Importantly, i27-Bregs also secrete i27-exosomes. We adoptively transferred *ex-vivo* generated i27-exosomes into mice and investigated physiological relevance of i27-exosomes during the CNS autoimmune disease, EAU. The data presented here provide unique insights into the biosynthesis and immunobiology of IL-27 cytokine and suggest that the i27-exosomes can be exploited as a stand-alone immunotherapy for CNS autoimmune diseases such as uveitis and multiple sclerosis.

## Results

### Characterization of i27-Breg and comparative analysis of i27-Breg and i35-Breg cells

**Figure 1:**
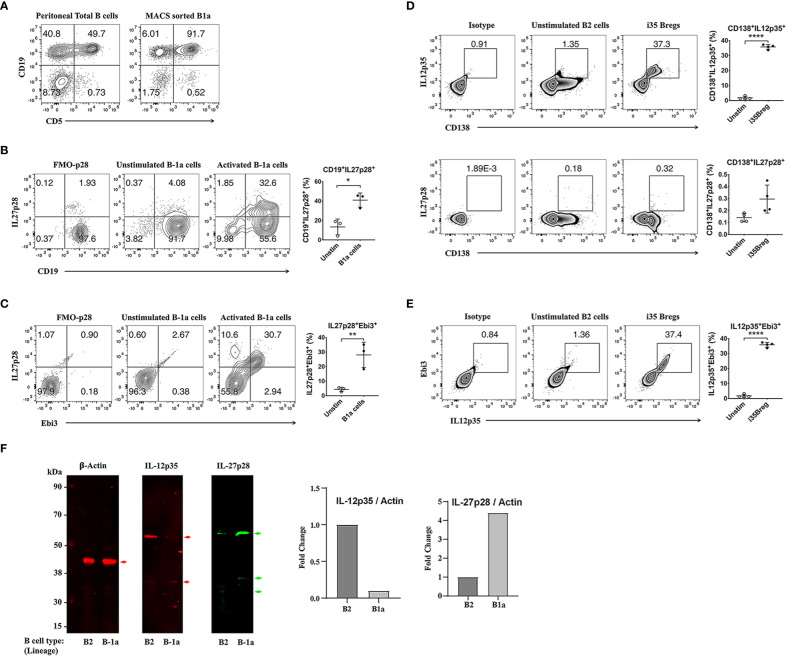
Characterization of i27-Breg and comparative analysis of i27-Breg and i35-Breg. **(A)** Primary mouse B cells isolated from the peritoneal cavity of C57BL/6J mice were magnetic-bead (MACS)-sorted using the B-1a cell isolation kit. Dead cells were excluded by FACS analysis using Fixable Viability dye eFluor 450 and top-right quadrants show percentage of CD19^+^CD5^+^ B-1a cells. (n=3/group) **(B)** Sorted B-1a cells were activated for 72 hours with anti-IgM/anti-CD40. Top-right quadrants and graphs show CD19^+^IL27p28^+^ and IL27p28^+^Ebi3^+^ B-1a cells **(C)**; (n=3/group). **(D, E)** B-2 cells were isolated from C57BL/6J mouse spleen and sorted with CD19^+^ magnetic beads. The cells were activated with anti-IgM/anti-CD40 for 72 hours, analyzed by FACS and dead cells were excluded by FACS analysis using Fixable Viability dye eFluor 450. Quadrants and graphs in **(D)** show CD138^+^IL12p35^+^ (top panel), CD138+IL27p28^+^ (middle panel) or CD138+IL12p35^+^Ebi3^+^ (Bottom panel) B-2 cells (n=4/group). **(F)** Western blot analysis of whole cell protein extracts of B-1a and B-2 cells. The data are presented as the mean ± SD of a representative dataset from at least three determinations, *p<0.05, **p<0.01, ****p<0.0001.

There are two B cell lineages in mammals: B-1 and B-2 cells. B-1 cells are the earliest B cells to arise during development and derive from fetal yolk sac or fetal liver and reside mainly in peritoneal cavity (PeC) or umbilical cord blood (CB) (21, 22). B-1 cell is characterized by expression of cell-surface CD5 on B-1a but not B-1b cells and polyreactive germline B cell receptor (BCR) with limited N-region diversity and they are considered as innate lymphocytes (23). In contrast, B-2 cells appear much later during embryonic development from the bone marrow. They reside mainly in lymphoid follicles or marginal zone of the spleen (MZ B cells) and follicular B cells are the largest Ag-specific B cell population in the periphery or tissues. Here, we describe isolation and characterization of the innate-like B-1a cells derived from cells of the B-1 lineage and show that they are functionally distinct from i35-Bregs of the splenic B2 lineage on basis of signature cytokines they produce. B-1a or B-2 cells were isolated from mouse peritoneal cavity or spleen respectively. The cells were stimulated with anti-IgM/anti-CD40 or LPS and dead cells were excluded by FACS analysis using Fixable Viability dye eFluor 450 and only live cells were analyzed. As shown, >90% of the CD5^+^CD19^+^ B cells were B-1a cells (**Figure 1A**) and ~30% of the BCR-activated B-1a cells express IL-27 (**Figures 1B, C**). On the other hand, ~37.0% of activated B-2 cells in the spleen were i35-Bregs while <1% produced IL-27 (**Figures 1D, E**). Thus, the highly purified B-1a cells utilized in this study preferentially produced IL-27 (i27-Bregs) but contained insignificant levels of IL-35-producing B cells (i35-Bregs). This observation is further confirmed by Western blot analysis of cells derived from B-1a and B-2 cells (**Figure 1F**).

### B-1a cells express membrane-bound IL-27

**Figure 2:**
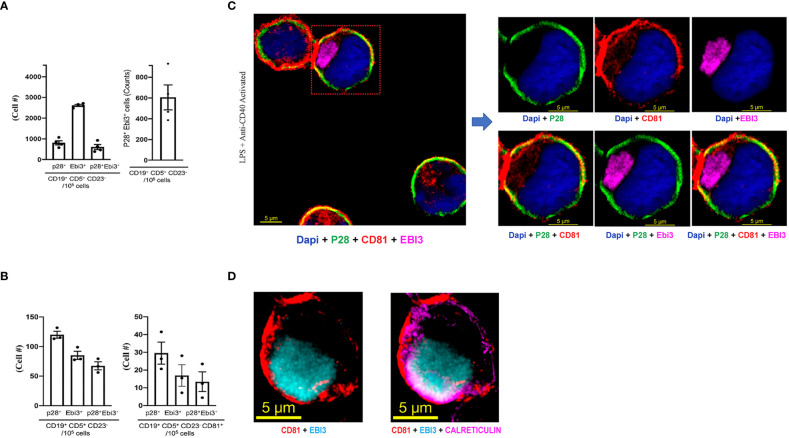
B-1a cells from the peritoneal cavity express membrane-bound IL-27. **(A)** B-1a cells from mouse peritoneal cavity were stimulated for 72 hours with anti-IgM/anti-CD40 and analyzed by intracellular cytokine staining assay. Graphs show absolute numbers of p28^+^, Ebi3^+^ or p28^+^Ebi3^+^ B cells in CD19^+^CD5^+^CD23^-^ compartment (left panel) and IL-27-producing B-1a cells (right panel) (n=3/group). **(B)** B-1a cells were stimulated for 72 hours with anti-IgM/anti-CD40. The B-1a cells were stained with p28, Ebi3 or CD81 antibodies and FACS analysis was performed without permeabilizing the cells. Left panel shows absolute numbers of CD19^+^CD5^+^CD23^-^ B-1a cells in the peritoneal cavity expressing IL-27 subunit proteins (p28, Ebi30 proteins). Right panel shows absolute numbers of B cells in CD19^+^CD5^+^CD23^-^CD81^+^ compartment that expressed p28 and/or Ebi3 on the B-1a plasma membrane(n=3/group). **(C, D)** Analysis and localization of cell surface expression of p28, Ebi3 or CD81 on activated i27-Breg cells by immunohistochemical staining and confocal microscopy (representative of three individual experiments).

i27-Bregs and i35-Bregs are thought to suppress inflammation through secretion of their signature cytokines, IL-27 and IL-35 respectively. However, mechanisms by which the p35 and Ebi3 subunits of IL-35 or p28 and Ebi3 subunits of IL-27 associate *in vivo* to form the functional IL-35 or IL-27 cytokine are unknown. Recently, i35-Bregs were found to secrete i35-exosomes that suppress neuroinflammation (17, 24). This observation is akin to the finding that IL-35-producing Treg cells (iT_R_35) display p35 on their cell surface and conferred regulatory functions to bystander T cells by coating them with IL-35^+^ extracellular vesicles (EVs) (24), suggesting that regulatory lymphocytes might express cell surface IL-35. In this study, we activated B-1a cells *in-vitro* for 72 hours by stimulation with anti-IgM/anti-CD40 and investigated whether the IL-27-producing B-1a cells express IL-27 on their cell surface or secrete exosomes that contain IL-27 (i27-exosomes). Intracellular cytokine expression analysis of the activated cells detected expansion of i27-Breg cells among CD19^+^CD5^+^CD23^-^, a cell phenotype that characterizes the B-1a lymphocyte lineage (**Figure 2A**). However, it was not clear whether the IL-27 detected by intracellular cytokine staining assay was on the B-1a cell surface or derived from the coating of the activated B-1a cells with IL-27^+^ EVs or i27-exosomes. Surprisingly, when we stained the B-1a cells with p28 and Ebi3 antibodies and then performed the FACS analysis without permeabilizing the cells, we detected p28 and Ebi3 expression on CD19^+^CD5^+^CD23^-^ and CD19^+^CD5^+^CD23^-^CD81^+^ cells, suggesting cell-surface expression of IL-27 on the B-1a cells and/or i27-exosomes (**Figure 2B**). The observation that IL-27 produced by B-1a cells might be membrane-bound is therefore surprising in view of the prevailing view that IL-27 is a soluble cytokine (4). To confirm that i27-Breg cells express membrane-bound IL-27, we cultured B-1a cells from the peritoneal cavity for 72 hours and performed immunohistochemical staining of the activated B-1a cells with antibodies specific to p28, Ebi3, as well as CD81, a tetraspanin protein which is also a BCR co-receptor (25). Visualization of IL-27 expression by confocal microscopy revealed colocalization of the p28 subunit to the plasma membrane in association with CD81 (**Figure 2C**). However, the staining pattern co-localized Ebi3 in the cytoplasm and endoplasmic reticulum (ER) with calreticulin, an ER resident protein or ER marker, suggesting that Ebi3 possibly localizes in lumen of the rough ER, with modest expression in the cytoplasm (**Figure 2D**; **Supplementary Video-1 Information**).

### B-1a cells secrete exosomes containing membrane-bound p28, Ebi3 and CD81

**Figure 3:**
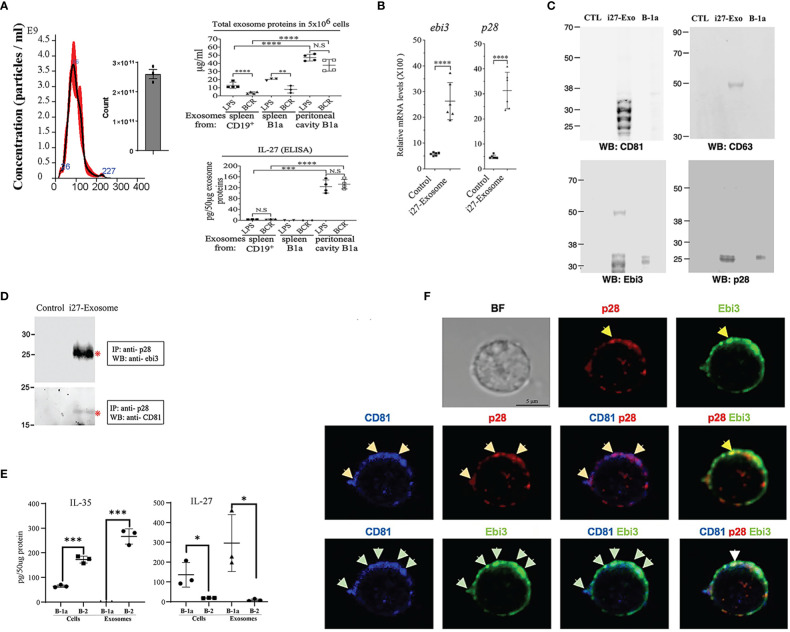
i27-Exosomes express membrane-bound p28, Ebi3 and CD81. **(A)** B-1a or CD19^+^ B-2 cells from mouse peritoneal cavity or spleen were stimulated with LPS or anti-IgM/anti-CD40 in exosomes-depleted medium for 72hrs (n=3-4/group). Exosomes in supernatant were purified and protein content in 5x10^6^ exosomes was quantified by BCA protein assay (top panel) and amount of IL-27 secreted (pg/50µg exosome protein) was determined by ELISA (bottom panel) (n=3-4/group). **(B-D)** Exosomes isolated from supernatant of the BCR-activated B-1a cells were subjected to qPCR analysis **(B)** (n=6/group), Western blot analysis **(C)** (n=1/group) or reciprocal immunoprecipitation analysis **(D)**. Controls in these analyses are exosome-depleted supernatant of B1a cell cultures. **(E)** Cell lysates and exosomes from activated B1a or B-2 cells were analyzed for IL-35 or IL-27 by ELISA(n=3/group). **(F)** For localization of IL-27 (p28 and Ebi3) and CD81 expression on the i27-Exosome membrane, the exosomes were captured with Tetraspanin Exo-Flow Capture Kit consisting of 9.1 µm diameter magnetic beads, stained with antibodies without fixation or permeabilization and then analyzed by confocal microscopy. The data are presented as the mean ± SD of at least three determinations, *p<0.05; **P < 0.01; ***P < 0.001; ****p<0.0001.

B-1a or B-2 cells were activated *in-vitro* by stimulation with LPS or anti-IgM/anti-CD40 (BCR) in exosome-depleted culture medium. EVs were isolated and purified using Exo-Quick-TC exosome precipitation solution and only EVs ranging in sizes between 50-150nm (exosomes) as determined by the nanoparticle tracking analysis (NTA) method (**Figure 3A**) were used in this study (17). Regardless of the stimulus used to activate the cells, substantial amounts of IL-27 containing EVs or i27-exosomes were detected in supernatants of B-1a cells derived from peritoneal cavity compared to similarly cultured B-2 or B-1a cells derived from the spleen (**Figure 3A**). This result was confirmed by ELISA (**Figure 3A**, lower panel). To further increase amounts of exosomes for our studies, the sorted i27-Bregs from the peritoneal cavity were further expanded (>75%) by an additional 3-days cycle of stimulation with anti-IgM/anti-CD40 in exosome-depleted culture medium as previously described (17). qPCR analysis of purified i27-exosomes from 6 independent EV preparations confirmed that exosomes from each of the preparations expressed Ebi3 and IL-27p28 transcripts (**Figure 3B**). Western blot analyses also show expression of p28 and Ebi3 subunit proteins as well as the exosome-markers, CD63 and CD81 (**Figure 3C**). We also show that the exosome markers are enriched in exosomes isolated from supernatant of activated B-1a cells but absent in exosome-depleted supernatants (**Figure 3C**). Thus, the exosome-depleted supernatant that does not contain p28 or Ebi3 served as control in some experiments. This is consistent with use of exosome-depleted supernatants as controls for exosome preparation used in human clinical studies (26) or in characterizing exosomes used in rat EAU studies (27). Reciprocal co-immunoprecipitation analysis using antibodies specific to Ebi3 or p28 confirmed that the i27-exosomes express IL-27 (**Figure 3D**). Consistent with the preferential production of IL-27 by B-1a cells but not B-2 cells, B-1a cells secrete i27-exosomes but not i35-exosomes and conversely, B-2 cells secrete i35-exosomes but not i27-exosomes (**Figure 3E**). To directly demonstrate that the i27-exosomes express cell-surface IL-27, we incubated the i27-exosomes with the Tetraspanin Exo-Flow Capture Kit consisting of 9.1 µm diameter magnetic beads attached with streptavidin and tetraspanin-biotin antibodies as recommended by the manufacturer. After capture of the i27-exosomes on the magnetic beads we stained the beads with fluorescence-conjugated anti-p28, Ebi3 or CD81 antibodies and visualized specific binding and co-localization of p28, Ebi3 and CD81 on the i27-exosomes captured on Exo-Flow beads by confocal microscopy (**Figure 3F**). Interestingly, we detected CD81 in association with p28 on the surface of the i27-Breg cell while we localized CD81 expression with both p28 and Ebi3 on the i27-exosomes.

### IL-27-Exosomes suppress experimental autoimmune uveitis

**Figure 4:**
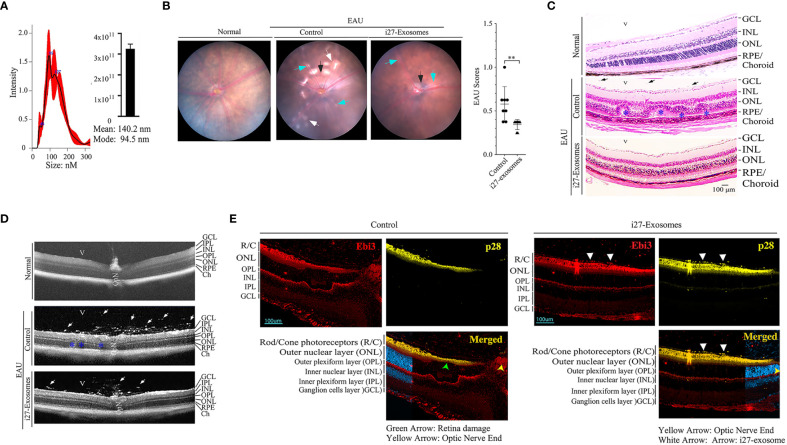
IL-27-expressing exosomes (i27-Exosomes) suppress experimental autoimmune uveitis (EAU). The exosomes used for treatment were isolated, ranged in size between 50 to 150nm and met criteria set by the 2018 International Society for Extracellular Vesicles (ISEV-18) for designation of EVs as exosomes **(A)** (n=3/group). Control for our EAU studies are exosomes isolated from resting unstimulated B-1a cells (Control-Exosome). EAU was induced in C57BL/6j mice by immunization with IRBP_651-670_ in CFA and disease progression was assessed by fundoscopy, histology and optical coherence tomography (OCT). **(B)** Representative fundus image of retina at day 14 after EAU induction reveal inflammation with blurred optic disc margins and enlarged juxtapupillary area (black arrows), retinal vasculitis (blue arrows) and yellow-whitish retinal and choroidal infiltrates (white arrows). Clinical scores and disease severity were based on changes at the optic nerve disc or retinal vessels and retinal and choroidal infiltrates (n=8/group). **(C)** Compared to mice treated with i27-exosome, histological images of day-21 eyes of mice that received the control exosome show substantial numbers of inflammatory cells in retina. H&E histological sections: Scale bar, 100µM. V, vitreous; GCL, ganglion cell layer; INL, inner nuclear layer; ONL, outer nuclear layer; RPE/CH retinal pigmented epithelial and choroid. Blue arrows, lymphocytes; Asterisks, retinal folds(n=8/group). **(D)** Representative OCT images show marked increase of inflammatory cells (white arrows) in the vitreous of mice treated with control exosomes(n=8/group). **(E)** For the analysis of IL-27 expression in retinas of EAU mice treated with exosomes, the mice were extensively perfused with saline solution and enucleated eyes were fixed in paraffin and IL-27 expression was detected by immunohistochemical analysis (white arrow heads). Retina pigmented epithelium (RPE); Photoreceptors (R/C); outer nuclear layer (ONL); outer plexiform layer (OPL); inner plexiform layer (IPL). The data are presented as the mean ± SD of at least three determinations, **P < 0.01.

We next investigated whether i27-exosomes can be used to suppress EAU, a T cell mediated autoimmune disease in mouse (13, 20). We induced EAU in WT C57BL/6J mice by active immunization with IRBP_651-670_ peptide in Complete Freund’s Adjuvant (CFA) and treated the mice with i27-exosomes. The control for our EAU studies are exosomes isolated from resting unstimulated B-1a cells (Control-Exosome). The exosomes ranged in size between 50 to 150nm, and met criteria set by the 2018 International Society for Extracellular Vesicles (ISEV-18) for designation of EVs as exosomes (**Figure 4A**). Mice received ∼2x10^10^ exosomes (20ng IL-27) by retro-orbital injection 6 days after EAU induction and every day thereafter until day 10 post-immunization as described (28). Clinical symptoms of EAU is generally manifested between post-immunization (p.i.) day-12 and day-22 (29) and we monitored progression and severity of uveitis during this period by fundoscopy, a non-invasive procedure that allows visualization of fundus (retina, macula, optic disc, fovea and blood vessels) using the ophthalmoscope or fundoscopy. Fundus images obtained on day-14 p.i. showclassical features of uveitis which include papillitis, blurred optic disc margins and enlarged juxtapupillary areas (black arrows); retinal vasculitis (blue arrows); yellow-whitish retinal and choroidal infiltrates (white arrows) (**Figure 4B**, left panel). On the other hand, the fundus images and bar graph of the clinical scores show a less severe disease in the eyes of the i27-exosome treated mice (**Figure 4B**, right panels). Histology of the day-21 retinas of control mice exhibit hallmark EAU features characterized by infiltration of large numbers of inflammatory cells into the vitreous, destruction of retinal cells and development of retinal in-folding (**Figure 4C**, top panel) while these pathological features were relatively mild in i27-exosome treated mice (**Figure 4C**, bottom panel). Optical coherence tomography (OCT) is a noninvasive procedure for visualizing retina microstructure. Analysis of the retinas by OCT shows substantial accumulation of inflammatory cells in the vitreous and optic nerve head of the control mouse retina (**Figure 4D**, top panel) but not the retinas of mice treated with i27-exosomes (**Figure 4D**, bottom panels). Non-Treg cells have recently been shown to propagate immune tolerance and suppress inflammatory responses by coating their cell-surface with IL-35-containing EVs secreted by Treg cells (24, 30). Because retinal cells suppress uveitis through production of IL-27, we examined whether the amelioration of EAU observed in this study derived in part from passive acquisition of i27-exosomes. EAU was induced, treated with exosomes as described above and the mice were sacrificed on day 22 p.i. at the tail end of the inflammatory response that coincides with disease recovery phase. The mice were extensively perfused with saline solution to remove blood from the central retinal artery or choriocapillaris and enucleated eyes were fixed in 10% formalin, sectioned and stained with p28- or Ebi3-specific antibodies. Immunohistochemical analysis of the sections revealed that IL-27 expression localized to the photoreceptor layer of the mice treated with i27-exosomes compared the untreated mice (**Figure 4E**). This observation is of interest because the photoreceptor cells are the primary site of tissue destruction during EAU while microglia cells in the ganglion cell layer are thought to be the main producers of IL-27 in the retina (13, 15). However, recent *in-vivo* imaging studies have revealed that microglia cells are transiently recruited to the photoreceptor layer in response to focal injury of photoreceptor cells and presumed to play a role to limit further damage to the affected photoreceptors (31). Moreover, the detection of IL-27 in the photoreceptor layer is consistent with previous reports of expression of high levels of IL-27 receptor by photoreceptors (32). Together, these results suggest that adoptive i27-exosome immunotherapy can confer protection against severe uveitis in mice. However, future studies are required to clarify whether the IL-27 detected in the photoreceptor layer derived from passive acquisition of i27-exosomes or i27-exosome-mediated upregulation of IL-27.

### Amelioration of EAU in mice treated with i27-exosomes correlates with inhibition of pathogenic Th1/Th17 cell responses and expansion of regulatory T cells

**Figure 5:**
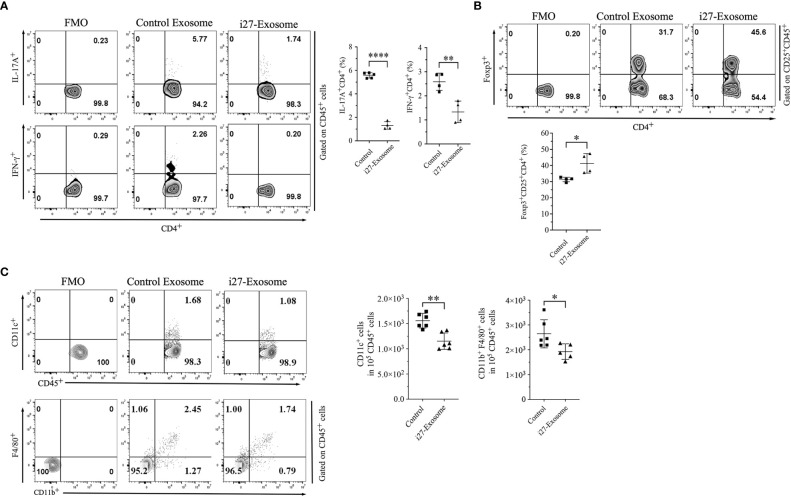
Amelioration of EAU in mice treated with i27-exosomes correlates with inhibition of pathogenic Th1/Th17 cell responses and expansion of regulatory T cells. Freshly isolated CD4^+^ T cells in the dLNs (axillary, cervical) of EAU mice were analyzed for expression of proinflammatory or immunosuppressive cytokines by the intracellular cytokine staining assay. Numbers in quadrants and the corresponding histograms indicate percentage of cells expressing IFN-γ and/or IL-17A **(A)** or Foxp3^+^CD25^+^ regulatory T cells **(B)** (n=4/group). **(C)** Cells isolated from the spleen were gated by FACS for myeloid cells and numbers in quadrants or the corresponding graphs indicate percentage of CD11c^+^ or CD11b^+^F4/80^+^ cells(n=4/group). The data are presented as the mean ± SD of a representative dataset from at least three determinations, *p<0.05, **p<0.01, ****p<0.0001.

Uveitis in mice is initiated by inflammatory cells that infiltrate the retina following the breakdown of the blood-retina-barrier and the pathogenic process is mediated primarily by Th17 and Th1 lymphocyte subsets (13, 33). We therefore investigated whether suppression of EAU observed in mice treated with i27-exosomes derived from inhibition of Th17 and/or Th1 cells by quantifying percentage of CD4^+^ T cells secreting the proinflammatory IL-17 and/or IFN-γ cytokines. Consistent with published reports, development of EAU in control mice was accompanied by significant increases of IL-17-expressing (Th17) and IFN-γ-expressing (Th1) cells in the draining lymph nodes (cervical and axillary lymph nodes) (**Figure 5A**). In contrast, we detected significantly lower percentages of IL-17-expressing and IFN-γ-expressing cells in mice treated with i27-exosomes (**Figure 5A**). In addition to its anti-inflammatory effects on proinflammatory lymphoid cells during EAU, we observed expansion of regulatory T cells in i27-exosome treated mice which correlated with amelioration of uveitis (**Figure 5B**). Although exact mechanism that induced expansion of regulatory T cells in i27-exosome treated mice is not clear, it is of note that i27-Breg immunotherapy ameliorates encephalomyelitis and uveitis in mice by propagating inhibitory signals that convert conventional B cells to regulatory lymphocytes secreting IL-10 and/or IL-35 in the eye, brain, or spinal cord (15). Moreover, in that study loss of IL-27Rα or IL-27 function abrogated capacity of i27-Bregs to suppress disease, suggesting critical role of IL-27 in the immune suppression (15). It is therefore conceivable that the IL-27 contained in i27-exosomes may promote differentiation and expansion of Tregs. Furthermore, decrease in percentages of CD11c^+^ or CD11b^+^F4/80^+^ myeloid cells in spleen of i27-exosome treated mice is also consistent with anti-inflammatory effects of i27-exosomes (**Figure 5C**).

### i27-exosomes upregulate expression of PD-1 and CTLA4 inhibitory receptors on T cells

**Figure 6:**
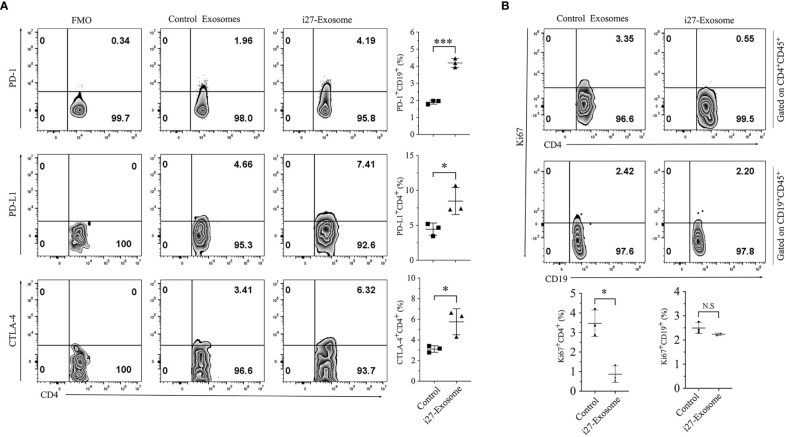
i27-exosomes-mediated suppression of T cell expansion during EAU correlates with the upregulation of PD-1 and CTLA4 inhibitory receptors. **(A, B)** Freshly isolated CD4^+^ T cells in the dLNs (Axillary, cervical) or B cells in the spleen of EAU mice treated with i27-exosomes (i27-exosomes) or mice with EAU treated with control exosomes. Cells were gated on CD4^+^CD45^+^ or CD19^+^CD45^+^ cells by FACS and analyzed for cell-surface expression of inhibitory receptors **(A)** or percentage of proliferating T or B cells using anti-Ki-67 antibodies (n=3-6/group) **(B)**. Numbers in quadrants and the corresponding histograms indicate percentage of cells expressing PD-1, PD-L1 or CTLA4 (n=3/group). The data are presented as the mean ± SD of at least three determinations *p<0.05, ***p<0.001.

Increase of cell-surface expression of inhibitory receptors such as PD-1 on B lymphocytes has been shown to enhance the survival and immune suppressive functions of Breg cells while upregulated expression of inhibitory receptors on T lymphocytes is associated with T cell exhaustion (34–36). We therefore investigated whether i27-exosomes suppressed uveitis by inducing checkpoint inhibitory receptors that promote exhaustion of autoreactive T cells. CD4^+^ T cells isolated from control or i27-exosome-treated EAU mice were reactivated *in vitro* and analyzed by FACS. Consistent with the immune-suppressive functions of i27-exosomes (**Figure 5A**), we observed significant increases of PD-1^+^, PD-L1^+^ and CTLA-4^+^ T cells in mice treated with i27-exosomes compared to controls (**Figure 6A**). Interestingly, effects of i27-exosomes are specific; while i27-exosomes induced significant reduction of the proliferative activities of T cells, they had no effects on B lymphocyte proliferation (**Figure 6B**).

## Discussion

The unique anatomic features of the eye and its sequestration from peripheral immune system provide a valuable framework for studying mechanisms that regulate inflammation in immune privileged sites like the CNS and have been valuable in validating efficacy of emerging therapies. Uveitis is a group of intraocular inflammatory diseases of infectious or autoimmune etiology. It accounts for more than 10% of severe visual handicaps which may include sight-threatening diseases such as Behçet’s disease, birdshot retinochoroidopathy, Vogt-Koyanagi-Harada’s, sympathetic ophthalmia and ocular sarcoidosis (37, 38). Immunosuppressive drugs such as corticosteroids, cyclosporin A, FK-506 or rapamycin and alkylating agents are standard of care depending on severity of uveitis (39, 40). However, prolonged use of these drugs increases risk of infections, malignancy, decreased lifespan and has been the impetus for developing alternative therapies for uveitis. It is therefore of note that in a recent preclinical study, i27-Breg immunotherapy suppressed encephalomyelitis and uveitis and ameliorated EAU and EAE without adverse effects, suggesting that autologous i27-Bregs might be a promising therapeutic approach for treating CNS autoimmune diseases such as uveitis and multiple sclerosis. However, a major obstacle to i27-Breg therapy is the difficulty of ascertaining the precise dose of IL-27 delivered in therapeutic settings.

In this study, we have shown that i27-Bregs which were isolated from the peritoneal cavity also secrete IL-27-containing exosomes (i27-exosomes). We show here that treatment of mice with i27-exosomes conferred protection against developing severe uveitis without provoking observable adverse effects. Compared to i27-Breg immunotherapy, i27-exosomes possess unique features that make i27-exosome immunotherapy a more effective and attractive therapeutic option. A quintessential feature of exosomes is that the exosome content is encapsulated by a lipid bilayer. Thus, in contrast to i27-Bregs, the i27-exosome cargo (e.g., IL-27p28 and Ebi3 subunits and/or RNA) is protected against degradation by proteases and nucleases and thus effectively delivered to the target tissue. Moreover, the IL-27p28/Ebi3 heterodimers confined in the same vesicle, obviating the dosing problem of determining the precise amount of bioactive IL-27 required to achieve a predictable therapeutic effect (17). Therapeutic use of i27-exosome also overcomes the technical difficulty associated with producing enough i27-Bregs for immunotherapy as >4x10^11^ exosomes can be isolated from a mouse and 2x10^10^ i27-exosomes can contain ~20ng IL-27. Furthermore, the blood-brain-barrier (BBB) and blood-retina-barrier (BRB) are impediments to the use of Breg cells as therapy for neuroinflammatory diseases. Because exosomes are nanosized vesicles (50-150 nm) that readily cross the BBB and BRB, i27-exosomes are efficient and ideal vehicle for delivering the immunosuppressive IL-27 cytokines to the immune privileged tissues of the brain and neuroretina.

Unlike proinflammatory members of the IL-12 family cytokines such as IL-12 and IL-23 that are secreted and function as disulfide-linked heterodimers, IL-27 is a non-covalently linked heterodimeric cytokine. Despite significant interest in IL-27 as a biologic for treatment of CNS autoimmune diseases, there is limited knowledge about its immunobiology or biosynthesis. An important unresolved issue relates to the mechanisms that initiate association of the IL-27p28 and Ebi3 to form stable heterodimeric IL-27 cytokine *in-vivo* and how stability or dissociation of the heterodimer is regulated under various physiological conditions. It is also not known whether the heterodimeric IL-27 cytokine or its single chain subunits play distinct role in proinflammatory or anti-inflammatory functions attributed to IL-27. Immunohistochemical and confocal microscopy analyses suggest localization of Ebi3 expression to the lumen of the rough endoplasmic reticulum (ER) while IL-27p28 co-localized on the i27-Breg plasma membrane in association with CD81, a tetraspanin protein that is also a component of BCR co-receptor complex. These results thus provide suggestive evidence that IL-27p28 might be a transmembrane protein while expression of Ebi3 in the ER is characteristic of secreted proteins. We propose that increased secretion of Ebi3 during inflammation might promote interactions of Ebi3 with the p28:CD81 complex which then sustain formation of transient membrane-bound IL-27 on i27-Breg cells. On the other hand, it is conceivable that reduced levels of Ebi3 in absence of inflammation may contribute to dissociation of Ebi3 from the IL-27p28:CD81 complex, thereby terminating immune suppressive activity of IL-27. Although the distinct patterns of IL-27p28 and Ebi3 expression provide insight into the biosynthesis of IL-27, structural analysis of the stoichiometry between Ebi3 and the IL-27 p28:CD81 complex on the B cell plasma membrane is required to better understand immunobiology of the biosynthesis of membrane-bound IL-27 utilized by i27-Bregs.

In summary, i27-exosome immunotherapy has several advantages over administration of IL-27 as a biologic or i27-Breg immunotherapy and they include: (i) overcoming technical difficulty of producing enough biologically active IL-27 or i27-Bregs for immunotherapy; (ii) encapsulation of IL-27 in a lipid bilayer confers protection from degradation and thus maintains integrity of IL-27 contained in the i27-exosome; (iii) confinement of IL-27p28 and Ebi3 in the same vesicle obviates the dosing problem of determining precise amounts of bioactive IL-27p28:Ebi3 heterodimer (IL-27) required to achieve therapeutic effect; (iv) capacity of i27-exosomes to cross the BBB and BRB makes i27-exosomes ideal vehicle for delivering immunosuppressive IL-27 cytokines to immune privileged tissues of the brain and retina. Interestingly, our investigation to determine whether i27-exosomes can be effective therapy for CNS autoimmune disease provided mechanistic insights into the biosynthesis of IL-27 and reveal that IL-27p28 is a transmembrane protein which interacts with Ebi3 to form the membrane-bound IL-27. However, the most important finding of this pre-clinical study is the demonstration that i27-exosomes ameliorates uveitis in mice, suggesting potential success of i27-exosome as a stand-alone therapy for human uveitis and other CNS neuroinflammatory diseases.

## Materials and methods

### Materials and data availability

Any requests for information on the materials used in this study or additional information required to reanalyze the data reported in this paper is available from the lead contact upon request.

Charles E. Egwuagu (egwuaguc@nei.nih.gov).

### Mice and ethics statement

Six- to 8-week-old C57BL/6J mice were purchased from Jackson Laboratory (Jackson Laboratory, Bar Harbor, ME). The mice were maintained and treated in accordance with National Eye Institute (NEI) and NIH Animal Care and Use Committee guidelines (Study # EY000262-19 & EY000372-14). All animal care and experimentation conformed to National Institutes of Health (NIH) guidelines and the experimental protocol was approved under NIH/NEI Animal Study Protocol (ASP) # NEI-597.

### Cell sorting and i27-Breg activation

Primary mouse B cells isolated from the peritoneal cavity or spleen of C57BL/6J mice were isolated by use of kits purchased from Miltenyi Biotec including B cell Isolation kit (130–090–862), CD19 MicroBeads (130–052–201) and B-1a Cell Isolation Kit (130–097–413). Sorted CD19^+^ or B-1a cells were stimulated with LPS (5µg per ml, Sigma), anti-CD40 Abs (10µg per ml; BioXcell) and anti-IgM Abs (5µg per ml; Jackson ImmonoResearch) for 72 hours as described (15).

### Experimental autoimmune uveitis

EAU was induced by active immunization of C57BL/6J mice with IRBP_651-670_-peptide (300µg per mouse) in a 0.2 ml emulsion (1:1 v/v with complete Freund’s adjuvant (CFA) containing 2.5 mg per ml *Mycobacterium tuberculosis* strain H37RA (2.5 mg per ml) sub-cutaneous as described previously (29). Mice also received *Bordetella pertussis* toxin (1µg/mouse) by intraperitoneal (i.p.) injection in 100 µl of RPMI 1640 medium containing 1.0% normal mouse serum on the same day of immunization. Mice received ∼2x10^10^ exosomes (20ng IL-27 per mouse) on day 6 post-immunization and every day until day 10 post-immunization by retro-orbital injection. For each study, 8 mice were used per group and they were matched by age and sex. Clinical disease was established and scored by fundoscopy and histology as described previously (16). Eyes were examined for disease severity using binocular microscope with coaxial illumination. Eyes for histology were enucleated 21 days post-immunization, fixed in 10% buffered formalin and serially sectioned in the vertical pupillary-optic nerve plane. All sections were stained with hematoxylin and eosin.

### Fundoscopy

Funduscopic examinations were performed at day 10 to 21 after EAU induction. Briefly, following systemic administration of systemic anesthesia [intraperitoneal injection of ketamine (1.4 mg/mouse) and xylazine (0.12 mg per mouse), the pupil was dilated by topical administration of 1% tropicamide ophthalmic solution (Alcon Inc., Fort Worth, Texas). Fundus image was captured using Micron III retinal imaging microscope (Phoenix Research Labs) for small rodent or a modified Karl Storz veterinary otoendoscope coupled with a Nikon D90 digital camera, as previously described (41). To avoid subjective bias, evaluation of the fundus photographs was conducted without knowledge of the mouse identity by a masked observer. At least 6 images (2 posterior central retinal view, 4 peripheral retinal views) were taken from each eye by positioning the endoscope and viewing from superior, inferior, lateral and medial fields and each individual lesion was identified, mapped and recorded. The clinical grading system for retinal inflammation was as established (41, 42).

### Imaging mouse retina by spectral-domain optical coherence tomography

SD-OCT is a noninvasive procedure that allows visualization of internal microstructure of various eye structures in living animals. An SD-OCT system with 820 nm center wavelength broadband light source (Bioptigen, NC) was used for *in vivo* non-contact imaging of eyes from control or EAU mice. Mice were anesthetized and the pupils dilated as described above. Mice were then immobilized using adjustable holder that could be rotated easily allowing for horizontal or vertical scan scanning. Each scan was performed at least twice, with realignment each time. The dimension of the scan (in depth and transverse extent) was adjusted until the optimal signal intensity and contrast was achieved. Retinal thickness was measured from the central retinal area of all images obtained from both horizontal and vertical scans from the same eye, using the system software, and averaged. The method used to determine the retinal thicknesses in the system software was as described (43).

### Proliferation assay

B-1a and CD4+ T cells were harvested from WT C57BL/6J mice. The B-1a cells were stimulated with anti-CD40 Abs (10µg per ml) and anti-IgM Abs (5µg/ml) while the CD4^+^ T cells were stimulated with anti-CD3 (2µg/ml)/anti-CD28 (2µg/ml) Abs. Both cell types were cultured in medium containing control or i27-exosomes. Proliferative capacity of either cell type was assessed by FACS using anti-Ki67 Abs.

### Flow cytometry

For intracellular cytokine detection, cells were stimulated for 4 h with PMA (20 ng per ml)/ionomycin (1 µM). GolgiStop was added in the last hour, and intracellular cytokine staining was performed using BD Biosciences Cytofix/Cytoperm kit as recommended (BD Pharmingen, San Diego, CA, USA). FACS analysis was performed on a CytoFLEX Flow Cytometer (Beckman Coulter, Indianapolis, IN, USA) using protein-specific monoclonal antibodies and corresponding isotype control Abs (BD Pharmingen, San Diego, CA, USA) as described previously (44). As FACS analysis was performed on samples stained with mAbs conjugated with fluorescent dyes, each experiment was color-compensated. Dead cells were stained with dead cell exclusion dye (Fixable Viability Dye eFluor^®^ 450; eBioscience), and only live cells were analyzed. Quadrant gates were set using isotype controls with less than 0.2% background.

### i27 exosome isolation

Isolation and purification of IL-27 producing B-1a cells (i27-Bregs) has previously been reported (15). Briefly, B cells isolated from mouse peritoneal cavity were activated with anti-IgM/anti-CD40 B-1a cells and non-B-1a cells were depleted by magnetic selection. The B-1a cells were then positively selected on CD5^+^ magnetic beads and we perform FACS analysis to confirm that i27-Bregs comprise >30% of the activated B-1a cells. We also routinely increase the percentage of i27-Breg cells by culturing the cells in exosome-depleted medium containing IL-27 (15). Supernatants from *in vitro* cultures of i27-Breg or control cells cultures are then subjected to sequential centrifugations at 300xg (10min), 3000xg (10 min) and 12000xg (10 min) and exosomes were isolated using the Exo-Quick-TC kit as previously described (17). For Western blot Analysis, the cleared supernatant was further subjected to ultracentrifugation at 100,000xg (2 h) followed by washing of the particulate fraction in PBS at 100,000xg (2 h). We measure exosome size distribution by Nanoparticle Tracking Analysis using the NanoSight system (Malvern Panalytical, MA, USA). Expression of exosome markers or IL-27 subunit proteins are then characterized and validated by Western blotting. Exosomes used in this study are isolated, purified and characterized according to recommendations of the International Society for Extracellular Vesicles (ISEV). Because B-1a cells are a relatively rare B cell population in the peritoneal cavity and each EAU study requires large amounts of exosomes, we routinely prepare several EV preparations which may contain varying amounts of i27-exosomes. For *in vivo* studies, we routine pool EV preparations isolated on the same day. The i27-exosomes are then characterized, quantified, aliquoted and stored frozen until use. We use the same batch of frozen i27-exosomes for each EAU experiment.

### Exosome RNA quantification

For EV RNA qPCR, total RNA was isolated from i27-exosomes isolated from the culture supernatant of activated B-1a cells and control exosomes from unstimulated B1a cells using SeraMir Exosome RNA Isolation and Purification Kit (System Biosciences, Palo Alto, CA, USA). The Kit provides Spike RNA for use as internal control.

### Immunofluorescence staining and confocal imaging analysis

B cells were activated as earlier described and adhered to glass slides. Briefly, the cells were fixed with BD-Fixation/permeabilization kit, adhered to glass slides by the Cytospin method or incubated for 30 min on Poly-L-lysine coated slide. Cells were blocked in 10% goat serum, 1% BSA, 0.1% Triton for 1 hour and in MOM blocking solution (Invitrogen) if the primary antibody is of mouse origin. Cells were then incubated with rabbit anti-CD81 (Cell Signaling), mouse anti-Ebi3 and rat anti-p28 (R&D system either MAB7430 or MAB18342). Cells were washed 3 time for 10 minutes and incubated with Alexa 488 (anti-rat) and Alexa 568 (anti-rabbit) and 647 (anti-mouse) secondary antibodies

### ELISA

IL-35 and IL-27 cytokine in cell lysate or purified exosomes protein lysate were quantified using mouse IL-35 and IL-27-specific heterodimeric ELISA kit (BioLegend), following the protocol as recommended by manufacturer.

### Immunoprecipitation and immunoblotting

Preparation of whole cell lysates and cleared lysates or cellular supernatants were immunoprecipitated with antibody that was pre-coupled to protein G-sepharose beads as described (45). Immunoprecipitates were resolved by SDS-PAGE and blots were probed with specific antibodies. The following antibodies were used for immunoprecipitation and/or Western blotting: p35(Abcam), p28 (Invitrogen), Ebi3, and β-actin (Santa Cruz, CA). Pre-immune serum was used in parallel as controls and signals were detected with HRP conjugated-secondary F(ab’)2 (Zymed Labs, San Francisco, CA) using ECL system (Amersham, Arlington Heights, IL).

### Statistical analysis

Graphs were plotted and analyzed using GraphPad Prism 9, two-tailed unpaired Student’s *t* test or One-way ANOVA with Tukey’s *post hoc* test depending on the experiments. Probability values of <0.05 were considered statistically significant. Data that is normally distributed are shown as mean ± SEM. Asterisks denote p value (*P < 0.05, **P < 0.01, ***P < 0.001, ****P < 0.0001).

## Data availability statement

The original contributions presented in the study are included in the article/**Supplementary Material**. Further inquiries can be directed to the corresponding author.

## Ethics statement

The mice were maintained and treated in accordance with National Eye Institute (NEI) and NIH Animal Care and Use Committee guidelines (Study # EY000262-19 & EY000372-14). All animal care and experimentation conformed to National Institutes of Health (NIH) guidelines and the experimental protocol was approved under NIH/NEI Animal Study Protocol (ASP) # NEI-597.

## Author contributions

MK, MY, C-RY, and EM performed EAU experiments and FACS analysis. MK, MY, and C-RY performed immunofluorescence and confocal analysis. EM, MY, and C-RY edited the manuscript. CE conceived, designed, supervised the project and wrote the manuscript. All authors contributed to the article and approved the submitted version.
